# Reform of care for people with mental illness in two German states before reunification: An oral history approach

**DOI:** 10.1177/0957154X251392921

**Published:** 2026-03-08

**Authors:** Georg Bornemann, Thomas Becker, Robert Feustel, Heiner Fangerau, Felicitas Söhner, Sven Speerforck

**Affiliations:** 1 Universität Leipzig, Germany; 2 Friedrich-Schiller-Universität Jena, Germany; 3 Heinrich-Heine-Universität Düsseldorf, Germany

**Keywords:** social participation, Germany West, Germany East, oral history, discourse theory

## Abstract

Between 1960 and 1985 efforts to reform psychiatry took place in the German Democratic Republic (GDR) and the Federal Republic of Germany (FRG). Although to differing degrees, these efforts showed parallel approaches, for example with regard to negotiations on coercion or the participation of mentally ill people. Oral history interviews conducted with contemporary witnesses from both states suggested changes in the direction of the reform of psychiatry and progress in social participation during the period. Contrasting historical sources with the interviews reveals a dissonance that can be understood in terms of discourse theory. Initial results indicate a convergence, across different political systems, toward improving social participation. Future research would benefit from cross reading this study’s results with other sources.

## Introduction

Psychiatric reforms in Germany have already been analysed and depicted from various perspectives for the period 1960 to 1985 (see e.g. Dörre, 2021; Hennings, 2015; Kersting, 2003). However, contemporary witnesses and their perception of the development of participation have rarely been included in these studies (Söhner et al., 2020). The same can be said of the reform actors’ memories of debates about civil rights and the associated issues of freedom and coercion. Reform processes were articulated in both German states from the 1960s onwards. It is questionable to what extent common goals, leitmotifs, ideas, and narratives can be constructed in the German Democratic Republic (GDR) and the Federal Republic of Germany (FRG). Comparisons against the background of different social systems could help identify factors critical to reform processes.

The situation of German psychiatry after the Second World War was precarious in several respects. In moral terms, many psychiatrists had participated in the National Socialist crimes against humanity, for example the sterilisation programmes, the “Aktion T4” and the ensuing decentral “euthanasia”, in which 200,000 patients with mental illness were systematically murdered (Hohendorf et al., 2002). In terms of the institutional infrastructure, hospitals were compromised by wartime damage to their physical plants, acute shortages of supplies, and an authoritarian spirit that Erwin Goffman would later refer to in describing “total institutions” (Fehlemann et al., 2017; Fangerau et al., 2021). Instead of a completely new beginning, post-war psychiatry was often characterised by personnel and conceptual continuities in both East and West Germany. Only in the GDR, which saw itself as anti-fascist, was there a more extensive (post-war) reappraisal of the murder of psychiatric patients during the Nazi regime (Hanrath, 2002).

In the 1960s and 1970s, mentally ill patients in the GDR and FRG were mostly still detained in large institutions rather than treated, resulting in long hospitalisations and insufficient prospects of recovery (Schulze and Rotzoll, 2018). It was not until the 1960s that greater reforms and reform initiatives were launched with the aim of improving psychiatric care. These developments took place in the context of international efforts to reform psychiatric care. Reform efforts can be traced back to the post-war period in almost all European countries (Bauer, 1991). As early as the 1950s, efforts were made in the UK to restructure large hospitals in favour of more outpatient services and the open-door principle. Similar efforts were made in the USA beginning in the 1960s. A notable development in France, which was later adopted by other countries, was the reorganisation of nationwide care into sectors from 1961 onwards. From the late 1960s and the 1970s in Italy, Franco Basaglia and colleagues fought successfully to abolish rigid and criminalising mental hospital structures, a move that attracted widespread international attention. The resulting mental health reform law was radical by international standards, with all Italian mental hospitals being closed from 1978 onwards and psychiatric care being redistributed to community services and general hospital psychiatric units (Schott and Tölle, 2006; Tansella, 1991).

There were also minor reform efforts in post-war Germany. Evidence of social psychiatric initiatives in the GDR date back to the 1950s. Examples include Dietfried Müller-Hegemann, who focused on restoring the ability to work as an important step in rehabilitation (Schmiedebach and Priebe, 2004). Or Ehrig Lange, who as clinic director in Mühlhausen-Pfafferode focused on open-door psychiatry (Felber and Sauermann, 2009). The latter also played a key role in a position paper following a symposium in Rodewisch in 1963 that can be considered the first structured reform effort in Germany after the Second World War (Rodewisch Theses: Renker, 1963). Rolf Walther, who also focused on a socio-psychiatric reorganisation of the clinic from the 1950s onwards (Hennings, 2015), managed the GDR district hospital in Rodewisch. The demands of the international symposium in Rodewisch, which was also attended by West German representatives, were:

- “active therapeutic approach” independent of diagnosis, course, and prognosis;
- combination of pharmacotherapy, occupational, and group psychotherapy;
- establishment of an aftercare system, creation of links between inpatient and outpatient care;
- Educational and public relations work on anti-stigmatization.

The Brandenburg Theses, which followed 10 years later, built on these demands, calling for improvements to psychotherapeutic care and to the “therapeutic community” (Renker, 1963; Schirmer et al., 1976). Historians have recognized the importance of these initiatives, but also that they had little lasting impact on nation-wide mental health care practices in the GDR (Kumbier et al., 2013). Neither these iniatives, nor the efforts undertaken at the University of Leipzig by Klaus Wiese, who, promoted open concepts and outpatient treatment, resulted in comprehensive reform of psychiatric care in the GDR (Steinberg, 2014). From the 1980s in particular, there was a lack of political will and financial resources (Steinberg, 2016).

By contrast in the BRD, the Psychiatry Enquête (1971–1975) was a democratically legitimised initiative launched by the federal parliament to systematically investigate the state of psychiatric care and to recommend reforms and improvements. Although there had already been calls for institutional reforms in the 1960s, it took the findings of the Enquête and the persuasive powers of the Christian Democratic member of parliament Walter Picard to convince the Bundestag of the need for change (Häfner, 2016; Söhner et al., 2018). The Enquête’s final report, which brought together the views of different schools of thought and professional organizations, was published in 1975 (Söhner, 2020).

The Enquête’s significance in transforming West Germany’s psychiatric landscape has been widely acknowledged in the academic literature (Brink, 2010; Brückner and Kersting, 2021; Dörre, 2021; Hanrath, 2002; Kersting, 1998). Both the final report and the process leading to it have been described as milestones in the reform of psychiatric care in the Federal Republic (Bühring, 2001). Although the government was at first reluctant, or at least not enthusiastic, about initiating reforms, the Enquête is credited with triggering a broad public debate about how society should deal with people suffering from mental illness (Falk, 2016). By revealing severe deficits and inhumane conditions in psychiatric institutions, the report sparked a broad discussion that contributed to a shift in public perceptions and greater acceptance of the mentally ill (Aktion Psychisch Kranke, 2001; Bühring, 2001).

Among other things, the commission’s final report called for the equal treatment of mentally and somatically ill patients and proposed a reorganisation of psychiatric care, shifting it from large institutions towards community-based care (Bundestag, 1975). These proposals had been influenced by the social psychiatry movement in West Germany and the politics of the late 1960s. Many progressive psychiatrists saw their professional calling to be not just to medical science but also to socio-political engagement. From their perspective, social determinants of illness and recovery had to be taken into account (Schmiedebach and Priebe, 2004). The Enquete served as a catalyst for their progressive agenda.

Although the Enquête and the new “Zeitgeist” triggered extensive restructuring that opened up mental health care and moved it into the community, psychiatric reform remained a work in progress (Bühring, 2001).

Although FRG and GDR took different paths in their attempts to implement reform, the evidence suggests that the exchanges between psychiatric representatives from both sides were lively and mutually significant (Beyer, 2018).

This was the case in spite of the GDR exerting political influence on higher education and medicine. Within psychiatry/psychology, in particular, officials attempted to promote Pavlov’s teachings and a Marxist-Leninist interpretation of medical science (Busse, 1998). Such attempts encounted the scepticism and outright opposition of many specialists. Contrary to state policy, there was no effective Soviet influence on the medical profession’s structure, mentality, and culture (Pasternack, 2015). However, other barriers prevented the free practice of science in the GDR. For example, a certain loyalty to the party line, also in the form of Socialist Unity Party (SED) membership, was a prerequisite for leading positions. Furthermore, travel to professional conferences in non-socialist countries was reserved for only a few people deemed sufficiently trustworthy by the regime (Wolle, 1999). Nevertheless, there is no evidence to support claims of systematic political abuse of psychiatry, as happened in the Soviet Union (Süß, 1998).

Despite the political differences, there were interdependencies and exchanges as well as common roots of psychiatric professionalism and practice. This applied to academia and scientific journals (with a wide distribution of some Western journals in the psychiatric institutions of the FRG and the GDR). Buttler and Steinberg (2024) discuss social psychiatric concepts developed in the FRG by Karl Peter Kisker at the Hannover Medical School and in the GDR by Klaus Weise and (the philosopher) Achim Thom at the Karl Marx University in Leipzig. Medicine, and in particular psychiatry, was relatively inert in the face of the different social conditions (Bornemann et al., 2025). Medicine’s proximity to long-standing bourgeois cultural, scientific, and elitist traditions appears to be relevant here (Hettling, 2015; Kocka, 2008). Thus, it is not surprising that the Enquête Commission’s recommendations were similar to the Rodewisch and Brandenburg theses.

Changes within medicine – and therefore psychiatry – are related to social and political developments. For example during the First World War, the frequent occurrence of war neuroses led to greater use of psychotherapeutic approaches and, as a result, psychotherapy gained in importance as a method used by neurologists (Bornemann and Steinberg, 2023; Schröder, 1995). Although such connections seem logical, the exact mechanisms governing the relationship between psychiatry and society are complex (Becker, 2024; Bornemann et al., 2024).

To this day, the inclusion, integration, and autonomy of people living with mental illness remain important issues that can benefit from studying earlier reform efforts (Kahl, 2016). Focusing on self-empowerment (Giertz et al., 2022; Konrad, 2021), we will use the concept of “social participation” to capture the aims of the respective reform efforts. Aspects of social participation had already been discussed during the Weimar Republic in what was then called “open welfare” (Hildebrandt, 1986). Although this term was not widely used in debates about psychiatric reform the 1960s and 1970s, integration into working life (outside sheltered workshops), housing options outside hospitals, subjectivation and opportunities for co-management in therapy can be subsumed unter this umbrella term.

In addressing the issues of social participation, integration, and the inclusion of mentally ill people, this study relies on the transcripts of oral history interviews conducted with mental health professionals whose careers spanned the era of psychiatric reforms (1960–1985) in the two German states.

## Methods: Oral history as a methodological approach to the history of psychiatry in the East-West context

This study uses the methods of oral history (see Lichtblau, 2021; Söhner et al., 2018; Sparing et al., 2024) to tap into the biographic perspectives of contemporary witnesses. It investigates oral sources and interprets them in the context of other written documents, targeting subjective experiences and recollections of historical events and structures. It accounts for the witnesses’ emotions and individual perspectives, which in turn reflect collective experiences (Plato, 1991, 2001; Söhner et al., 2024; Te Heesen, 2021; Wierling, 2003). The “thinking, feeling, and willing” of the contemporary witnesses is thus moved to the centre of interest (Dilthey, 1927). This approach helps to shed light on the perspectives of the marginalised groups who are often underrepresented in traditional written sources (Hansson et al., 2022). In this way, existing historical narratives and their respective interpretations of historical events can be scrutinised and re-evaluated (Hoyle, 2022; Niethammer, 1985; Söhner, 2024).

Twenty semi-structured interviews were conducted with individuals based on their experiences between 1960 and 1985. This period was chosen to coincide with the first psychiatric reforms in Germany, which date from the 1960s, but also to exclude the later years of the so-called peaceful revolution. Although Mikhail Gorbachev’s glasnost and perestroika exerted a profound influence on East German society in particular, these developments lie outside this study’s focus on the pre-1985 landscape of psychiatric reform.

Given the historical and thematic contours of the study, interviewee selection followed the logic of theoretical sampling (Glaser and Strauss, 1967). Drawing on the qualitative and inductive-deductive research strategies used by oral historians (Corbin and Strauss, 1990; Glaser and Strauss, 1967; Mayring, 2016), each interview was treated not merely as a narrative source, but also as an analytically valuable case. Adopting a hermeneutic-epistemological approach, the interviews were understood to be inherently open-ended, helping to generate new hypotheses and research perspectives. The sampling process thus strove for theoretical-empirical saturation: similar cases were incorporated in order to substantiate recurring patterns, while contrasting perspectives were intentionally included to sharpen conceptual distinctions. Further sampling was considered complete once inferential saturation was reached, i.e. when no novel thematic insights emerged from additional interviews (Glaser and Strauss, 1967).

To evaluate the adequacy of our sample, we drew on the concept of information power, as proposed by Malterud et al. (2016). This concept, which shifts the analysis from numerical sufficiency to the relevance and depth of the information yielded, reinforced our assumption that twenty interviews, if well-chosen, could be sufficient to ensure robust qualitative insight.

The focus of this inquiry was exclusively on the recollections of people working inside (or outside) mental health services during the study’s time frame. The interviews did not include people with mental illnesses or with personal experience of using psychiatric and other public services at the time. The study therefore reflects a work-related professional retrospective on the issue of participation.

To reflect the East-West dynamic intrinsic to our research, interviews were conducted with individuals from two regions of comparable size (each with roughly five million inhabitants before German reunification in 1989/90):

- for the GDR, the former districts of Leipzig, Dresden, and Karl-Marx-Stadt (now Chemnitz), which largely correspond to today’s federal state of Saxony, and
- for the FRG, the administrative district of Düsseldorf.

These regions were also chosen due to certain reformist initiatives: on the one hand at the hospital in Rodewisch and the Psychiatric University Hospital in Leipzig, on the other hand in the Rhineland Regional Association (LVR) and the Rhineland Society for Social Psychiatry (RGSP).

Roughly the same number of people were interviewed from both regions and respectively from different psychiatric (e.g. doctors, nurses, psychologists) and non-psychiatric fields (e.g. church, politics, business, culture). An anonymised list of the interviewees’ age, gender, and profession can be found in the supplemental material. The structured interviews were designed to address central topics of the research and to allow for associative reminiscence and personal narratives (Helfferich, 2022). Thematic focal points included:

- the type of professional activity, its setting, processes, formative figures;
- the opportunities to contribute criticism or suggestions;
- the positively or negatively connoted or omitted topics of discussion;
- the perceived interactions between psychiatry and society;
- the professional and/or private contacts in the respective “other” Germany, as well as memories about East-West tensions.

The interviews were conducted between October 2023 and April 2024. Although explicit questions about participation were not included in the interview guidelines (available in the supplemental material to this article), the topic was repeatedly raised by both GDR and FRG interviewees. Accordingly, this article focuses on interview sequences that relate to the participation, integration, and inclusion of people with mental illness, as well as to the broader project of opening psychiatry, both within and beyond institutional settings. The material was organised thematically to identify intersecting discourses and recurring narrative patterns, with interpretive frameworks visualised through qualitative content analysis (Mayring, 2014).

All interviews were conducted either individually or in pairs by the authors, recorded, and subsequently transcribed in full. The transcripts were analysed using a mixed inductive-deductive coding approach (Stamann et al., 2016), drawing on the established principles of qualitative content analysis (Mayring, 2014). The coding process was informed by the logic of theoretical sampling, in line with qualitative traditions that prioritise typification and conceptual refinement over statistical generalisability (Mayring, 2016). Initially, an extensive matrix was created that categorised the data by interviewee and question. The transcripts were then examined repeatedly in order to identify emerging themes; these were discussed, reduced to essential interpretive categories, and continually refined (Corbin and Strauss, 1990).

To facilitate a rigorous and transparent analytical process, the software MAXQDA 24 (VERBI Software; Berlin, Germany) was employed. A coding framework was developed based on discussions of key themes within the research team. Throughout the coding process, the researchers remained in close contact in order to ensure consistency, address discrepancies, and enhance interpretive validity. Inter-coder comparison and communicative validation within the research group—as well as in the Düsseldorf Oral History Group (Düsseldorfer Forschungswerkstatt Oral History)—contributed additional analytical depth, enabling the team to clarify distinctions and commonalities across the thematic landscape.

To preserve the anonymity of participants while enabling demographic contextualisation, each quotation was asigned a coded identifier (e.g. IPW6, IPO2). Since both the interviews and the analysis were conducted in German, full translations into English were deliberately avoided to minimise distortions in meaning. However, selected quotations were translated into English using a near-literal, one-to-one approach, in order to ensure accessibility for international readers without compromising the integrity of the source material.

## Results

### On coercion and freedom

According to the interviewees, the psychiatric practices in the 1960s and 1970s that gave rise to grievance included many forms of coercive treatment—for example involuntary commitment, forced medication, or restraint. Negotiations about the scope and legality of coercive measures and the associated debate about freedom are a central and recurring element in the memories of the specialists:

It’s not a problem to keep someone quiet until early if the police are still holding them [. . .]. But early on you have to resolve the conflict and also have to deal with the aggression that has happened to the person. (IPO1)^1^

Bad events were, for example, when patients could not be calmed down [. . .] when they had to be forced. [. . .] And I have to say, we all suffered from that too. Because we didn’t want it. And actually the patients sometimes said afterwards: ‘We knew you didn’t want it’. But sometimes we had to fixate. (IPW7)

The interviewees certainly do not consider coercion to be only a therapeutic tool. But they are aware of the consequences associated with its use in human interaction. Exploiting the power imbalance between practitioner and patient, which could be a significant factor in addition to the original, medico-therapeutic justification, is remembered as being a risk, a possibility, but not a rule. The interviewees remember experiencing the tension between coercion and professional authority, including issues of recognition and respect.

So the question of these open doors [. . .] has to do with freedom. [. . .] And I think the debate about freedom is actually one of the most existential debates in psychiatry. (IPW6)

This controversy involving the infringement of civil rights is reflected in efforts to avoid coercive measures by enhancing communication with the patient. A group of progressively orientated social psychiatrists who made non-violence their credo reported the use of this approach, especially in the FRG:

Open-door psychiatry, as little medication as possible, no violence. Those were the guiding principles [. . .] (IPW2)

In the seventies, we managed with very little violence. But as doctors, we also talked a lot with the patients. So sometimes we sat down for an hour or more and tried to make sure that someone stayed and didn’t have to be forced to stay. (IPW6)

According to the interviewees’ recollections, psychiatric practice without the use of force or coercion was a leitmotif of the reform efforts, but ultimately it was not feasible. In both the GDR and the FRG, it was closely related to specific situations and individuals:

You then knew exactly, nurse X, carer Y, he does it by force and she does it with the tongues of angels, yes? Sometimes that works, sometimes that works. (IPO1)

According to the recollections, communication was increasingly being used more consciously as the means of choice. The interview excerpts suggest that there was a trend, over time, of practitioners devoting significant additional effort to avoiding coercion. This shift underscores the idealistic approaches adopted by a “new” generation of psychiatrists. The simultaneous pharmacological progress of the 1960s and 1970s met with approval: antipsychotics and sedatives opened up additional possibilities of countering psychomotor agitation without physical violence.

That time when it was all about psychopharmaceuticals and neuroleptics and so on, when you could immediately alleviate a very acute situation of anxiety. And you didn’t have to put the nets on or anything like that, which no longer existed when I started. (IPO2)

Nevertheless, some of the interviewees also recalled a critical examination of the use of powerful medications, questioning circumstances under which their use was ethically responsible:

The observation of psychotropic drugs, the effect. Because we also sometimes felt: Oh, they act like a straitjacket. [. . .] Yes, that always received critical scrutiny, a review: What can we change and what do we need to change? (IPW3)

These reflections point to a broader cultural negotiation within psychiatry regarding the legitimacy and limits of coercive practice. Notably, the excerpts not only report on institutional conditions, but frame coercion within a moral economy of suffering, responsibility, and professional learning. The language employed by interviewees—speaking of “bad events”, “we all suffered”, or “tongues of angels”—reveals an effort to humanise their role and mitigate guilt. In some cases, these narratives appear to offer retrospective justification, often embedded in emotionally resonant anecdotes or imagined patient responses. Such patterns suggest that coercion is not remembered as a neutral procedure but as a deeply ambivalent act that required personal reconciliation.

What remains striking is that coercion is rarely described in concrete procedural detail. The absence of specifics—such as the exact forms or thresholds of restraint—may point to a kind of narrative silencing or to a discursive discomfort that persists even in retrospective accounts. In this sense, the interviews serve not only as historical testimonies but as moral texts that reflect how professionals narrate and process ethical tension. The remembered shift from physical to communicative control, and the cautious embrace of pharmacological tools, reflect the reformist ethos of the period. However, the ambivalence surrounding the use of both coercion and medication remains palpable.

From a discourse-analytical perspective, one might read these interviews as expressions of a professional field negotiating its transition: from custodial to communicative, from paternalistic to participatory, from physical to pharmacological control. Yet even within these narratives of progress, there is a lingering awareness of unresolved contradictions—a psychiatry that aspires to humanism, but remains structurally exposed to the realities of power, control, and institutional inertia. As such, these memories illuminate not only individual experience, but the moral fault lines of psychiatric reform itself.

### Opening psychiatry to society

The position of psychiatry as an institution in society becomes clear in the memories of representatives from the former GDR and FRG who have no psychiatric background:

And then in Düsseldorf there is a state hospital in Grafenberg. And Grafenberg is a neighbourhood [. . .] actually. But when it was said that someone had come to Grafenberg, then everyone knew straight away [. . .] they had come to the state hospital, right? [. . .] It was a synonym for that [. . .] And something like that was associated with fear and horror. [. . .] It was really associated with a kind of prison. (IGW3)

But the fear of being sent to a psychiatric ward, so to speak, [. . .] was great. [. . .] So I think, on the whole, many people didn’t have much confidence in psychiatry. (IGO3)

Outsiders often see psychiatry as a frightening unknown. The image of a prison implies a place of repression where inmates/patients are incarcerated and where outsiders have no access, neither physical or otherwise. The debate about “open doors” appears significant, not only in the context of patients’ rights, but also with regard to opening up psychiatric institutions. Specialists in both the GDR and FRG recalled groundbreaking debates in this regard:

The thing is, psychiatry can’t seal itself off. Society, which is outside, brings it in, right? (IPW4)

And then it’s always been about the question: openness of the institution, making psychiatry as open as possible. So the media basically supported the path to open psychiatry and were behind it. Politicians too. Not quite as long as the media. (IPW6)

There was always an endeavour to have an impact on the outside world. I can also remember, although I no longer have any material about it, we were also encouraged by our boss to always write an article in the newspaper about psychiatric issues, suicide or depression or something like that. So we always tried to make the wall around psychiatry more permeable. (IPO2)

Making psychiatry more visible to the outside world is remembered as a key concern, both in the GDR and the FRG. However the impact of public debate on psychiatry, be it on either side of the inner-German border, tends to be denied.

I don’t believe that society had any influence on psychiatry. Or I can’t remember it playing a role. But that’s also because the whole field of psychiatry was, to a certain extent, hived off. (IPO2)

For the period 1960–1985, hospital staff paint a picture of psychiatry in a state of flux: socio-political issues hardly ever penetrate directly from outside, but there is an endeavour to soften the isolation. A psychiatrist who worked in the GDR, on the other hand, saw an advantage in the isolation of the speciality:

This may sound strange now, but I experienced psychiatry as, and this was also an aspect of liking to go into psychiatry for me, I experienced psychiatry more as an extraterritorial area where you were left alone. Where the state had little or no influence. Patients could say whatever they wanted about what was unruly, politically unruly. It didn’t matter. (IPO4)

Of course, there was no freedom of the press in the GDR. However interviewees emphasised that in certain respects psychiatry offered a space to escape political control, even though it was never possible to entirely evade political considerations. The political dimension of psychiatry also played a role in the FRG under different circumstances:

And then I went into psychiatry at the end of the sixties [. . .]. And that was a political decision. So I thought that psychiatry is the medical speciality where you can have the greatest social impact [. . .] (IPW6)

Given psychiatry’s isolated post-war standing within medicine and society, not least due to the popular stigma associated with mental illness, a new generation of politically motivated specialists set out to break through their self-perception of this isolation. According to contemporary witnesses, psychiatry and related topics became more visibly localised within society. In retrospect, mental health professionals not only see themselves as having been involved in treating people with mental illness but also envisage their work as related to a socio-political mission.

Although no direct influences of emergent public criticism of psychiatry are reported, the impression remains that shifts in common values could have indirectly seeped into the institution. Critical, progressive voices changed the social climate and thus also the perspectives of actors working in the psychiatric contexts.

These recollections suggest that the institutional boundary between psychiatry and society was perceived as both rigid and permeable—a paradox that shaped the professional self-understanding of the interviewees. The image of psychiatry as a “walled” or even “extraterritorial” space recurs in various forms, sometimes serving as a site of critique, sometimes as a zone of protected autonomy. Particularly in the GDR, this isolation could be framed not merely as a constraint but as a space of possibility, where political interference was perceived as less intense than in other medical domains. Yet this autonomy was ambivalent: it protected practitioners and patients from external ideological pressure, but it also entrenched institutional seclusion and hindered broader social engagement.

The efforts to “make the wall more permeable”, whether through public communication, open-door policies, or cultural outreach, reflect a growing awareness of psychiatry’s social responsibility. At the same time, they reveal the limits of what was discursively possible within the professional and political frameworks of the time. The denial of direct societal influence on psychiatric practice—voiced by several interviewees—should not be mistaken for a lack of impact. Rather, it reflects a form of professional distancing that may have served to preserve a sense of integrity in a politically charged environment. It is precisely in this tension—between societal embeddedness and professional autonomy—that the modernisation of psychiatry was negotiated.

What emerges is a dynamic in which psychiatry simultaneously resists and responds to societal impulses. While the interviews rarely name concrete political reforms as catalysts, they testify to a slow but palpable shift in cultural attitudes—what might be described as a quiet convergence between professional introspection and external moral expectations. The “opening” of psychiatry was thus not merely spatial or architectural, but discursive: a process through which new forms of talking about, practising, and legitimising psychiatry entered the institutional repertoire. In this light, the period between 1960 and 1985 appears not as a rupture, but as a phase of discursive reconfiguration—a gradual, and at times contradictory process of reintegrating psychiatry into the social fabric.

### Psychiatry and participation

Interviewees from both the FRG and GDR recalled specific programmes that enabled patients to participate in social life (outside of psychiatry) or that were intended to educate and inform the wider public about psychiatry:

In this depression group [. . .] we had an afternoon for relatives once a week, right? We drank coffee with them and ate a few biscuits. And that also had a positive effect, right? (IPW4)

The next stage [. . .] was to involve family members in the therapeutic processes. At that time, this was called bifocal therapy. Groups of family members were formed. Groups of married couples were formed, and so on. (IPO2)

So we had patient clubs [. . .] They served as venues [. . .] free of charge. Patients went dancing there. As young colleagues, we danced with the patients. [. . .] So it was fraternisation on a small scale. (IPO1)

The softening of the boundary between psychiatry and the outside world certainly had therapeutic aims (family groups/“bifocal therapy”). However, this opening up was not solely driven by medical interests. Rather, the interviewees focussed on socio-political motivations. Their work was no longer regarded solely as that of medical professionals; instead, it was also intended to facilitate social participation (in GDR and FRG interviews).

At the time, the psychiatric clinic had ten tickets to the municipal theatres. The tickets were given to the patients, who then together went to the theatre or the opera or somewhere else. And that was the idea that we could overcome the boundary, the abnormal barrier between the institution and society or between the sick and the healthy or presumably healthy, that you could overcome these boundaries. (IPO2)

When I [was] on the ward on weekends, [. . .] I had conversations with patients or read aloud to them[. . .] or I organised a theatre visit. So I very much worked in the area of leisure activities. (IPW3)

A specific feature of the GDR that repeatedly appears in interview sequences is the integration of patients into industrial work processes.

Work therapy [. . .] was also an issue. At the time, semi-industrial work therapy was introduced. And, [. . . it was done so] without any influence from industry as far as standards and so on were concerned. (IPO4)

Rehabilitation legislation [. . .] obliged the company to [reserve] 10 per cent of its positions for rehabilitating patients. That came in the eighties. (IPO1)

The second level was then [. . .] to publicise this rehabilitative idea, that if someone from the company was on sick leave due to schizophrenia, the job was retained until they returned (IPO2)

According to contemporary witnesses, in both the GDR and the FRG patient care was no longer seen as isolated, monolithic, or institutional, but instead increasingly focused on patients’ everyday lives.

To think of community psychiatry not only in terms of the clinic, but to develop an entire system of community psychiatric facilities. (IPW2)

And when I started in 1973, I came to a clinic that had had a psychiatric outpatient clinic for many years, that had already had a day clinic for seven years, that had partly open wards, and that was differentiated into admission wards and discharge wards. It was also differentiated according to certain diagnostic specialisations. (IPO4)

As we tried to do through [patient] clubs, [. . .] we always provided special care for a city district before sectorisation came. (IPO2)

In view of structural reforms and tendencies towards open psychiatric care, interviewees recalled changes in everyday ward routines and communication. Patients were remembered as being increasingly involved in decision-making, while symbols of the power imbalance (such as doctors’ coats) were abolished in some places and jobs were created for mentally ill patients not only in industry but also in hospitals.

If we have such seriously ill people now, we have to change the atmosphere. And we can only change the atmosphere by employing sick people who have the perspective of those affected. (IPO1)

The boss was more of a primus inter pares. [. . .] We could talk about everything. [. . .] The patients too. We were in the ward meeting, then a patient attacked a doctor because of her behaviour. [. . .] or anything that didn’t go well, patients could talk about it. (IPW3)

For example, our boss [. . .] was a person who had an anthropological approach to things, so to speak. He said: ‘Gown off, gown off. That the patient communicates with you on a different level’, That was good thinking. (IPO2)

From these and similar sequences of interviews, it is clear that patients were no longer perceived by practitioners solely as objects of treatment, and the perspectives of patients were increasingly valued and listened to. In summary, interviewees recalled a wide range of changes that promoted patient participation, including patient groups and jointly attended cultural events. Community-based restructuring of care and increasing patient participation in everyday ward routines were obvious. There was, in this respect, no difference between GDR and FRG interviewees. Only labour-related rehabilitation measures were specific to the GDR.

This comparative account of psychiatric participation in both the FRG and the GDR reveals a shared trajectory towards deinstitutionalisation and social integration, albeit shaped by differing socio-political frameworks. What stands out is the evident shift from a paternalistic, medically dominated model towards a more participatory, socially embedded approach to mental health care. In both contexts, the traditional barriers between “patient” and “healthy” were actively challenged, not merely through clinical innovation but through the deliberate fostering of social interactions and community involvement.

The GDR’s distinct emphasis on labour integration, embedded in its socialist economy and legislation, illustrates how political ideology directly influenced rehabilitation policies. The introduction of mandated rehabilitation jobs and semi-industrial work therapy reflects an institutional commitment to maintaining the patient’s social and economic role, thus reinforcing the state’s broader collectivist goals. Conversely, the FRG’s initiatives centred more on community psychiatry infrastructure and cultural participation, reflecting a pluralistic and increasingly decentralised welfare state.

Crucially, the abolition of hierarchical symbols – such as doctors’ coats – and the inclusion of patients in decision-making processes signal a profound cultural transformation within psychiatric institutions themselves. This aligns with broader social movements in the 1970s advocating for empowerment and patient rights, underscoring the permeability between psychiatric reform and wider societal changes.

Ultimately, these narratives reveal how psychiatric practice became a site of socio-political contestation and transformation. Patient participation emerged not only as a therapeutic goal but also as a mode of social emancipation, challenging the stigmatising binaries of illness and normality. This evolution in psychiatric care reflects a fundamental redefinition of the patient’s role—from a passive recipient to an active agent within both the clinical setting and the social world.

## Discussion

Caution is required when interpreting the interviews. Given the many parallel narratives, it is important to discuss a number of limitations related to how humans frame their memories: Most of the contemporary witnesses interviewed were oriented towards social psychiatry and reform. This bias reflects personal preferences, affiliations and networks. The similarities observed with regard to the opening up of psychiatry and issues of patient participation suggest that professional boundaries (and conflicts) in psychiatry did not run along the inner-German border (Beyer, 2018) but instead along professional orientations (or “schools”, e.g. biological psychiatry vs. social psychiatry). The interview material is also likely to have been shaped by today’s views of the past, including our own focus on participation and a teleological framework that influenced the recall and “production of memories”. Despite the many insights elicited from the interviews, their historical significance is limited. This is not so much to draw skeptical conclusions from the claims of contemporary witnesses, as it is to underscore an important methodological reason for taking interview contents not as the goal, but as the starting point for additional historical inquiry.

Any conclusions are further limited by the absence of the voices of service users. This study provides a work-related professional perspective mostly from mental health care practitioners. To arrive at a more complete picture, the same level of attention would need to be devoted to the recollections of patients/service users, specifically their “take” on (and experience of) all aspects of participation.

Furthermore, several classic historiographic questions arise. What impact did the progressive impulses outlined above have in terms of the structures of psychiatric care in the districts and more generally national contexts? Are we dealing with exceptions expressed in the (specific) interviews, that is, with changed practices that were unusual and ahead of their time? Or did the reform efforts remembered follow a slowly emerging trend? The interviews tend to suggest the latter. However, it would not be surprising if representatives of social psychiatry had played more of a pioneering role, while the wider need for reform was delayed and slow to reach the general public. This pioneering spirit was evident, for example, in the questioning of clinic structures or in the form of participatory patient work (e.g. Schirmer et al., 1974).

It could therefore be useful, with the help of other sources, to reconstruct a more precise chronology of the events. This could help explain historical changes and provide insights into the intertwined relationship between historical events and structures (Koselleck, 1979). Phenomena that are negotiated are directly integrated into structures that both limit and enable them. At the same time, the same events can help reveal dynamics that change structures. When it comes to the relationship between civil society or the public on the one hand and psychiatric practice and its institutions on the other, the interplay between historical structures and events is likely to be of particular interest.

In the interview transcripts of this study, the stories shared from East and West reveal a striking similarity of debates and practices. Contemporary witnesses referred to elements of a cultural inclination towards change. This is remarkable in that the interview guide was exploratory and only referred to “psychiatric reform”. No explicit question addressed the opening of psychiatry or issues of participation. However, the topic appeared to be relevant to interviewees in remembering the development of psychiatry from the 1960s to the 1980s. Aspects of participation and openness constitute a consistent narrative in the interviews. This includes the identifiable focus on dealing with violence and coercion. We have identified a number of excerpts in the transcripts that indicate that social participation was an explicit focus and goal in the practice of psychiatric care in both the FRG and the GDR from the 1960s to the 1980s. Given that the two German states were fundamentally different in terms of their socio-economic, political, and cultural organization, it is striking to note the similarities in their narratives. In view of their shared traditions until 1945, one way of reflecting on the parallels lies in a shared (professional, institutional, philosophical-humanistic, and general) history. This suggests that the narratives reflect a transnational professional discourse of a gradual and constant movement towards an opening of institutions (albeit with the parallel persistence of the “old” psychiatric institutions).

However, in certain respects, the shared stories are inscribed in opposing constellations. In the East, a bottom-up process can be observed from the Rodewisch theses to the Brandenburg theses and the various endeavours reported in the interviews. Practical work on the ward fueled more or less powerful impulses that aimed to open up and humanise psychiatry as a whole. In the West, on the other hand, a top-down process was channelling events, with a state institution (the Enquête Commission) addressing an obvious and indisputable problem and proposing changes. In any case, future research will need to clarify more precisely how the respective dynamics have affected the structures as a whole and what role the efforts to open and humanize psychiatry outlined in the interviews have played in this.

These relationships between the practices of progressive awakening reported in the interviews and the structural environment in which they take place have another dimension. Beyond typical historiographic questions, it is possible to ask about the narrative structures of the interviews and their discursive embedding. All of the interviews analysed so far testify to an historically new medico-ethical self-awareness in dealing with patients and running institutions. The contemporary order of medical and psychiatric discourse, with its ethical imperatives, can be felt everywhere, the lack of alternatives to the reforms of that time is undisputed, and the self-reflection of the profession, which remains necessary today, is a discursive prerequisite for speaking.

However, the theoretical consideration that personal memories are influenced and sometimes even overwritten by altered “discursive formations” (Foucault, 1969) is not entirely absurd. “The symbolic order”, to which memories inevitably belong, “is characterised by a specific mode of causality”, which Slavoj Žižek describes as “retroactive causality”. While “[p]ositive, ‘substantial’ causality [. . .] is linear-progressive”, “the cause precedes its effect”, this is reversed in the symbolic order, that is, in the discursive network from which we cannot escape. The “symbolic effectiveness” (i.e. the discursive effects and attributions of meaning) “consists in a continuous ‘rewriting [of] one’s own past’, in that [. . .] past significant traces are included in new contexts”, “which retroactively change their meaning” (Žižek, 1994: 57). In other words, the selection, framing, and ultimately the meaning of historical events is never self-evident. Rather, the symbolic network of the present determines how, when, and why events are considered historically significant and thus worth remembering and mentioning. In the mode of retroactive causality, this coding, which is never apolitical or value-neutral, creates to a certain extent the events that are considered relevant, together with their contexts of justification, which then characterise memory.

Applied to the topic and material of this article, this would mean analysing the interviews in terms of discourse theory and contrasting them with other narrative sources of the period in question. How have the possibilities of what can be said shifted? More specifically: Can a narrative difference be identified when it comes to the need for reform and its justification? Which positions that may have been contentious or even marginalised at the time have now become fixed assumptions? Such discursive shifts, which we can only speculate about here, could have led to the reform processes being remembered differently in a retroactive way. These considerations emphasise the need for further research. In this context, it is essential to expand the approach to include contemporary, written sources, which will enable the oral sources presented here to be categorised (and put in their contemporary contexts) to gain discourse-theoretical insights into the negotiation of processes within psychiatry.

## Supplemental Material

sj-docx-1-hpy-10.1177_0957154X251392921 – Supplemental material for Reform of care for people with mental illness in two German states before reunification: An oral history approach Supplemental material, sj-docx-1-hpy-10.1177_0957154X251392921 for Reform of care for people with mental illness in two German states before reunification: An oral history approach by Georg Bornemann, Thomas Becker, Robert Feustel, Heiner Fangerau, Felicitas Söhner and Sven Speerforck in History of Psychiatry

## Notes

1. All contemporary witness quotes were translated from German into English by the authors.
