# Supplementary material for: Reform of care for people with mental illness in two German states before reunification: An oral history approach
Source: Hist Psychiatry. 2026 Mar 8;37(2):193–210. doi: 10.1177/0957154X251392921 (PMC13237210; doi:10.1177/0957154X251392921)
Supplement: sj-docx-1-hpy-10.1177_0957154X251392921 – Supplemental material for Reform of care for people with mental illness in two German states before reunification: An oral history approach [file sj-docx-1-hpy-10.1177_0957154X251392921.docx]

# Supplement

## Interview guide

**Discussion BEFORE recording:**

Information about the project. Information on data protection, recording/use/archiving of the interview. Clarification of open questions. Signing of consent form. Information that consent can be withdrawn at any time without giving reasons. Information that the recording can be interrupted at any time. Any further questions? If not, then start recording.

**Active in non-psychiatric fields**

You worked or were active in one of the following areas in the years 1960-1985:

Press/ media/ newspapers, adult education, trade unions/ company groups, church groups, cultural institutions or initiatives

You did this either in the Rhineland or in Saxony. We are grateful for your cooperation. In this preliminary study, we want to investigate the question of whether (remembering the time mentioned) there were any indications of connections between topics or “discourses” in society (particularly in your area of activity at the time) and topics or “discourses” in psychiatry. We call discourses complexes of topics that are discussed in a social system (society, psychiatry) and which are (also) reflected in written testimonies/documents/media products, e.g. newspaper or other texts. It is important for us as researchers that you relax and do not limit yourself to “perfect” or ‘complete’ or “meaningful” memories. Let your memory play a little freely. We open with a total of nine open questions:

1. Would you please tell us about your activity or activities (professional employment or other, voluntary activity) (in the years 1960-1985)? Would you please also describe the institution, the setting, the team in which you worked? Were there any formative academic colleagues/mentors?
2. How do you remember the key points/structure/process of a day in the setting/task area you described? What tasks did you have in your environment?
3. What do you remember as special events in your work during this period?
4. Do you remember topics/debates that were repeatedly raised/discussed/debated in your work/in your institution/with your colleagues?
5. What opportunities did you have to voice criticism (or suggestions)?
6. Do you remember topics of discussion from the time that you remember (in conversation with other people in your work or activity environment) as being important and particularly unpleasant or stressful or negative?
7. Do you remember topics from that time that seemed important to you, but which you do not or never remember being discussed at the time? So-called Blind spots
8. Did topics relating to the psyche or psychiatry play a role in your field of activity?
9. were you in contact with or did you maintain professional exchange with colleagues in the “other Germany” (East-West)? Do you spontaneously think that the East-West tension played a role in connection with the topics/discourses that were discussed in your work at the time?
10. We have now discussed all the points we have considered. Are there any other aspects that you think are important and that you would like to add?

**Psychiatry professionals**

You worked in psychiatry between 1960 and 1985. You did this either in the Rhineland or in Saxony. We are grateful for your cooperation. In this preliminary study, we want to investigate the question of whether (remembering the period in question) there were any indications of connections between topics or “discourses” in society and topics or “discourses” in psychiatry. We call discourses complexes of topics that are discussed in a social system (society, psychiatry) and which are (also) reflected in written testimonies/documents/media products, e.g. newspaper or other texts. It is important for us as researchers that you relax and do not limit yourself to “perfect” or ‘complete’ or “meaningful” memories. Let your memory play a little freely. We open with a total of nine open questions:

1. Would you please tell us about your activity or activities (professional employment or other voluntary activity) (in the years 1960-1985)? Would you please also describe the institution, setting, team in which you worked? Were there any formative academic colleagues/mentors?
2. How do you remember the key points/structure/process of a working day? What tasks did you have in your environment?
3. What do you remember as special events in your work during this period?
4. Do you remember topics/ debates that were repeatedly raised/ discussed/ debated in your work/ in your institution/ with your colleagues?
5. What opportunities did you have to voice criticism (or suggestions)?
6. Do you remember topics of discussion from that time that you remember (in conversation with others in your work or activity environment) as being important and particularly pleasant or positive?
7. do you remember topics from that time that seemed important to you, but which you do not or never remember being discussed at the time? Sog. Blind spots
8. were you in contact with or did you have professional exchanges with colleagues in the “other Germany” (East-West)? Do you spontaneously think that the tension between East and West played a role in the topics/discourses that were discussed in your work at the time?
9. do you remember topics that had an “external” impact on your work in psychiatry, i.e. from everyday life/society “into psychiatry”?
10. We have now discussed all the points we have raised. Are there any other aspects that you think are important and that you would like to add?

## Anonymized list of interviewees with regard to age, gender, profession and region

**West – District of Düsseldorf**

IGW 1 female, *1958, librarian

IGW 2 female, *1949, teacher, Lord Mayor

IGW 3 female, *1957, sociologist

IGW 4 male, *1961, musicologist, journalist

IPW 1 male, *1942, hospital chaplain

IPW 2 male, *1941, psychiatrist

IPW 3 female, *1948, sociologist

IPW 4 male, *1945, nurse

IPW 5 male, *1945, head of the health department

IPW 6 male, *1941, psychiatrist

IPW 7 female, *1941, nurse

**East – Former districts of Leipzig, Dresden, Karl-Marx-Stadt (Chemnitz); Saxony**

IGO 1 male, *1950, mechanical engineer, miner

IGO 2 female, *1956, mechatronics engineer, city council

IGO 3 male, *1937, theologian

IGO 4 male, *1950, musician

IPO 1 male, *1950, psychiatrist

IPO 2 male, *1937, psychiatrist

IPO 3 female, *1964, nurse

IPO 4 male, *1948, psychiatrist

**Not usable due to the geographical restriction**

NN male, *1954, teacher
